# The therapeutic potential of psilocybin in depression resistant to psychotropic drugs

**DOI:** 10.1192/j.eurpsy.2023.1767

**Published:** 2023-07-19

**Authors:** J. Ramírez González, I. Fernández Márquez, S. Ferreiro González, E. M. Ruíz

**Affiliations:** 1 Psychiatry, Consorci Sanitari de Terrassa, Barcelona; 2 Psychiatry, Consorci Sanitari de Terrassa, Barcelina, Spain

## Abstract

**Introduction:**

The use of hallucinogens has accompanied the human being throughout history. In the 1970s, studies focused on the therapeutic potential of hallucinogens were blocked due to their misuse in the young population. At present, psilocybin is re-emerging as the center of attention due to its possible therapeutic potential in different psychiatric pathologies such as depression, anxiety or substance use.

**Objectives:**

The main objective of this work has been to review recent studies on the therapeutic potential of psilocybin in drug-resistant depressive disorder.

**Methods:**

For the search for articles, the search strategy “psilocybin AND depression” was established in PUBMED. Regarding the inclusion criteria, it was established that they were recent articles, in Spanish or English and that the full text was freely accessible. On the other hand, those articles whose studies did not focus on humans and resistant depressive disorder were excluded. A total of 19 articles were obtained to review.

**Results:**

Focusing on Drug-Resistant Depressive Disorder, multiple studies have agreed that the administration of one or two microdoses (10-25mg) of psilocybin accompanied by psychotherapy improves the clinical picture for at least 6 months. These results make us feel optimistic in the search for new treatments in the field of mental health.

**Conclusions:**

Psilocybin microdoses associated with psychotherapy improves depressive symptoms in a patient resistant to common antidepressants.

The psilocybin response in terms of improvement of the depressive symptoms persists after 6 months of evolution.

One or, in some two cases, two microdoses of psilocybin (10-25mg) are enough to obtain statistically significant results in the improvement of the depressive symptoms.

**Disclosure of Interest:**

None Declared

